# Heterogeneous nuclear ribonucleoproteins R and Q accumulate in pathological inclusions in FTLD-FUS

**DOI:** 10.1186/s40478-019-0673-y

**Published:** 2019-02-12

**Authors:** Lauren M. Gittings, Sandrine C. Foti, Bridget C. Benson, Priya Gami-Patel, Adrian M. Isaacs, Tammaryn Lashley

**Affiliations:** 1 Department of Neurodegenerative Disease, UCL Queen Square Institute of Neurology, University College London, London, UK; 2 Queen Square Brain Bank for Neurological Disorders, Department of Clinical and Movement Neurosciences, UCL Queen Square Institute of Neurology, University College London, 1 Wakefield Street, London, WC1N 1PJ UK; 3 UK Dementia Research Institute at UCL, UCL Queen Square Institute of Neurology, University College London, London, UK

**Keywords:** FUS, Heterogeneous nuclear ribonucleoprotein, hnRNP R, hnRNP Q, FTLD, Frontotemporal lobar degeneration

## Abstract

Frontotemporal lobar degeneration (FTLD) is pathologically subdivided based on the presence of particular pathological proteins that are identified in inclusion bodies observed post-mortem. The FTLD-FUS subgroup is defined by the presence of the fused in sarcoma protein (FUS) in pathological inclusions. FUS is a heterogeneous nuclear ribonucleoprotein (hnRNP) protein and a member of the FET (FUS, EWS, TAF15) protein family. It shuttles between the nucleus and cytoplasm, and has been implicated in many cellular functions including translation, splicing, and RNA transport. EWS, TAF15 and the nuclear import receptor transportin have been shown to co-accumulate with FUS in neuronal inclusions specifically in FTLD-FUS, with transportin-positive inclusions most frequently observed. Here, we report the identification of hnRNP R and hnRNP Q in neuronal cytoplasmic and intranuclear inclusions in the frontal cortex and hippocampus of FTLD-FUS patients, as frequently as transportin. hnRNP R and hnRNP Q were not found in the characteristic pathological inclusions observed in FTLD-TDP (subtypes A-C). Additionally, we studied the expression of hnRNP R in the frontal and temporal cortices from patients with FTLD and found significantly increased expression of the heterogeneous nuclear ribonucleoprotein R in several FTLD disease groups. Our identification of the frequent presence of hnRNP R and hnRNP Q in FTLD-FUS inclusions suggests a potential role for these hnRNPs in FTLD-FUS pathogenesis and supports the role of dysfunctional RNA metabolism in FTLD.

**Electronic supplementary material:**

The online version of this article (10.1186/s40478-019-0673-y) contains supplementary material, which is available to authorized users.

## Introduction

Frontotemporal lobar degeneration (FTLD) is a broad term used to describe the major pathology underlying a clinically heterogeneous group of neurodegenerative diseases characterised by progressive changes in executive function, behaviour and/or language. Macroscopically, FTLD is typically identified by significant atrophy of the frontal and temporal cortices of the brain, while microscopically, the disease is characterised by the presence of abnormal intracellular protein aggregates. FTLD can be pathologically sub-divided into three major groups based on the main protein species identified in pathological inclusions; tau (FTLD-Tau), the TAR DNA-binding protein 43 (TDP-43) (FTLD-TDP) or the fused in sarcoma (FUS) protein (FTLD-FUS) [28, 35, 36]. Each group can be further sub-categorised based on the types of inclusions present. The FTLD-FUS group encompasses three pathological diagnoses; neurofilament inclusion body disease (NIFID), basophilic inclusion body disease (BIBD) and atypical frontotemporal lobar degeneration with ubiquitinated inclusions (aFTLD-U), collectively accounting for approximately 5–10% of all FTLD cases [27, 33, 39, 41, 42, 55]. A commonality among all three is the presence of pathological inclusions containing the FUS protein.

FUS is a multi-functional 53 kDa DNA/RNA-binding protein, belonging to the FET protein family. These are highly conserved, nuclear proteins that are ubiquitously expressed and are involved in various aspects of DNA and RNA metabolism, including RNA processing, transcription, splicing, transport and DNA repair [2, 26, 29, 54]. FET proteins are able to shuttle continuously between the nucleus and cytoplasm via the interaction between their non-classical nuclear localisation signal, PY-NLS, and the nuclear import protein, transportin 1 (TRN1) [24, 58, 59], which has been shown to label all FUS positive inclusions [6]. In addition to FUS, the two other members of the FET protein family, Ewing’s sarcoma (EWS) and TATA-binding protein-associated factor 15 (TAF15), have also been found to label a proportion of pathological inclusions in FTLD-FUS [40]. This observation has led to the hypothesis that disruption of the nuclear import of FET proteins by TRN1 may be contributing to pathogenesis in FTLD-FUS [43].

However, it is not only FET proteins that have been identified as components of pathological inclusions in FTLD-FUS cases. Our group previously reported several other RNA binding proteins are present to varying degrees within these inclusions following pathological and biochemical analysis of the heterogeneous nuclear ribonucleoprotein (hnRNP) family of proteins in FTLD-FUS cases [17]. Prompted by the fact that, in addition to being a FET protein, FUS can also be classified as an hnRNP (hnRNP P2) [7], a screen of 11 hnRNPs indicated the infrequent presence of hnRNP D, G, I and L and the more frequent presence of the TRN1 cargo, hnRNP A1, in FUS-positive neuronal cytoplasmic and intranuclear inclusions [17].

hnRNPs are a large family of proteins, which can shuttle between the nucleus and cytoplasm to carry out a variety of functions linked to nucleic acid metabolism, including nuclear (transcription, splicing, 5′ capping and polyadenylation) and cytoplasmic (mRNA transport, stability, translation and degradation) functions [15, 20]. The different hnRNPs frequently have overlapping functions and often perform their functions as part of a larger co-operative protein complex, however they also have individual specialised roles that are dependent on specific RNA-protein or protein-protein interactions [15, 20]. Given that hnRNPs perform a diverse range of functions linked to RNA metabolism, the presence of various hnRNPs in FUS inclusions implies that the pathogenesis of FTLD-FUS extends beyond FET proteins, TRN1 cargoes and dysfunctional nuclear import, and implicates a wider dysregulation of RNA binding protein metabolism. Here we provide further evidence to support this hypothesis by reporting the presence of two additional hnRNP proteins – hnRNP R and hnRNP Q - in pathological inclusions specifically in FTLD-FUS.

We used immunohistochemical, biochemical and expression analysis to investigate a role for hnRNP R in FTLD. Due to the sequence homology between hnRNP R and hnRNP Q we investigated the presence of both proteins in the pathological inclusions of FTLD-FUS and undertook a semi-quantitative assessment of pathological inclusions containing both hnRNP R and hnRNP Q compared to inclusions containing FUS and/or TRN1. We found hnRNP R and hnRNP Q to be present in essentially all FUS inclusions, indicating a potential role in FTLD-FUS pathogenesis.

## Materials and methods

### Cases

Brains were donated to the Queen Square Brain Bank for Neurological Disorders (UCL Queen Square Institute of Neurology) and the Medical Research Council London Brain Bank for Neurodegenerative Diseases (Institute of Psychiatry, King’s College, London). The demographic and clinical data of all cases used in this study are listed in Table 1. FTLD-FUS cases used in this study had previously been pathologically diagnosed as NIFID (*n* = 6, cases 1–6) or aFTLD-U (*n* = 7, cases 7–13) and have been previously reported [27]. FTLD-TDP cases used included FTLD-TDP A (*n* = 19), FTLD-TDP B (*n* = 3), FTLD-TDP C (*n* = 7) and neurologically normal controls (*n* = 6). Ethical approval for the study was obtained from the Local Research Ethics Committee of the National Hospital for Neurology and Neurosurgery.

**Table 1:** Case demographics of cases used in the study

Cases	Disease group	Age at onset	Age at death	Disease duration	Gender	Post-mortem delay (hours)
1	FTLD-FUS	41	43	2	F	55
2	FTLD-FUS	44	46	2	M	96
3	FTLD-FUS	63	59	6	F	2
4	FTLD-FUS	43	46	3	F	30
5	FTLD-FUS	69	72	3	F	90
6	FTLD-FUS	66	69	3	F	102
7	FTLD-FUS	49	55	6	F	3.5
8	FTLD-FUS	43	53	10	F	96
9	FTLD-FUS	55	58	3	F	N/A
10	FTLD-FUS	40	51	11	M	12
11	FTLD-FUS	44	51	7	M	24
12	FTLD-FUS	47	53	6	M	5
13	FTLD-FUS	51	60	9	M	48
*FTLD-FUS summary*		*50 (9.5)*	*55 (8.5)*	*5.5 (3)*	*5 M:8F*	*47 (40)*
14	FTLD-TDP A	66	74	8	F	86
15	FTLD-TDP A	43	45	2	M	26
16	FTLD-TDP A	53	63	10	M	77
17	FTLD-TDP A	62	68	6	M	99
18	FTLD-TDP A	58	67	9	F	115
19	FTLD-TDP A	56	67	11	F	85.5
20	FTLD-TDP A	57	62	5	F	63
21	FTLD-TDP A	66	71	5	M	52
22	FTLD-TDP A	58	66	8	F	107
23	FTLD-TDP A	47	53	6	M	34
24	FTLD-TDP A	53	61	8	M	72.5
25	FTLD-TDP A	57	62	5	M	93
26	FTLD-TDP A	62	72	10	M	97.5
27	FTLD-TDP A	49	55	6	M	29
28	FTLD-TDP A	75	78	3	F	36
29	FTLD-TDP A	83	87	4	F	69
30	FTLD-TDP A	57	63	6	F	85
31	FTLD-TDP A	72	79	7	F	50
32	FTLD-TDP A	62	68	6	F	100
*FTLD-TDP A summary*		*59 (10)*	*66 (10)*	*6.6 (2.4)*	9 M:10F	*71 (28)*
33	FTLD-TDP B	67	69	2	M	70
34	FTLD-TDP B	63	67	4	F	45.5
35	FTLD-TDP B	63	83	20	F	45
*FTLD-TDP B summary*		*64.3 (2.3)*	*73 (8.7)*	*8.6 (9.8)*	*1 M:2F*	*53.5 (14)*
36	FTLD-TDP C	58	73	15	F	38
37	FTLD-TDP C	59	73	14	F	84
38	FTLD-TDP C	64	78	14	M	27
39	FTLD-TDP C	64	74	10	M	19
40	FTLD-TDP C	50	65	15	M	52
41	FTLD-TDP C	61	66	5	M	71
42	FTLD-TDP C	44	67	23	M	76
*FTLD-TDP C summary*		*57.1 (7.5)*	*70.8 (4.9)*	*13.7 (5.5)*	*5 M:2F*	*52 (25)*
43	Control	80	N/A	N/A	M	16
44	Control	79	N/A	N/A	F	89
45	Control	80	N/A	N/A	F	49
46	Control	93	N/A	N/A	F	30
47	Control	73	N/A	N/A	F	24
48	Control	83	N/A	N/A	F	20
Control summary		*81.3 (6.6)*	*N/A*	*N/A*	*1 M:5F*	*14 (3.5)*

### mRNA expression analysis

Total RNA was extracted from the frontal and temporal cortices of FTLD-FUS (*n* = 5), FTLD-TDP A (*n* = 19), FTLD-TDP B (*n* = 3), FTLD-TDP C (*n* = 7) and normal controls (*n* = 6) using the Qiagen RNeasy kit. 100 ng of total RNA from each sample was analysed using the NanoString nCounter analysis system (Nanostring Technologies, Seattle, WA) using a pre-designed codeset, which has been previously reported [17]. The codeset contained probes for detection of the gene of interest; *HNRNPR*. Probes were designed according to the manufacturer’s design principles [18]. The laboratory running the assay was blinded to case diagnoses, and samples of cases or controls were randomly assigned to plates to avoid run-order bias. Raw counts were subjected to a technical normalization and normalized to the geometric mean using nSolver Analysis Software v2.0 (NanoString). Biological normalization was performed using reference genes (*CLTC, GAPDH, GUSB, HPRT1, PGK1, and TUBB*) included in the codeset. Statistical analysis of was performed using GraphPad Prism 5 software.

### Immunohistochemistry

Seven-micron-thick paraffin-embedded frontal cortex and hippocampal sections were cut from the cases listed in Table 1. Sections were deparaffinised in xylene and rehydrated using graded alcohols. Endogenous peroxidase activity was blocked with 0.3% H_2_0_2_ in methanol followed by pressure cooker pre-treatment in 0.1 M citrate buffer, pH 6.0. Non-specific binding was blocked with 10% dried milk solution. Tissue sections were incubated in the relevant primary antibody for 1 h at room temperature, or overnight at 4 °C. The following primary antibodies were used: FUS (Novus, aa1–50, 1:200), TRN1 (Abcam, ab1303, 1:500), hnRNP R (Abcam, ab30930 1:200), hnRNP Q (Thermo PA5–15009). Tissue sections were incubated with the relevant biotinylated secondary antibody (DAKO, swine anti-rabbit, 1:200, or goat anti-mouse, 1:200) at room temperature for 30 min, prior to incubation with avidin-biotin complex (ABC; DAKO) for 30 min. Antibody binding was visualised by 3,3 di-aminobenzidine, activated by H_2_O_2_, and cell nuclei were counterstained with Mayers haemotoxylin.

### Double-label immunofluorescence

Seven-micron-thick paraffin-embedded frontal cortex and hippocampal sections were sequentially co-stained using rabbit anti-FUS (Novus Biologicals NB100–565, 1:200) and goat anti-hnRNP R (Santa Cruz sc-16,541, 1:200) primary antibodies. After appropriate pre-treatment, tissue sections were incubated with the FUS primary antibody overnight at 4 °C, followed by incubation with a swine anti-rabbit secondary (Dako,1:200) for 1 h at room temperature, prior to a 30 min incubation with ABC. FUS binding was visualized using TSA fluorescein amplification kit (Perkin-Elmer). Tissue sections were then incubated with goat anti-hnRNP R primary antibody overnight at 4 °C, followed by incubation with a mouse anti-goat secondary antibody for 1 h at room temperature, prior to a 30 min incubation with ABC. Binding of the second antibody was visualized using TSA Cyanine 3 amplification kit (Perkin-Elmer). Cross reactivity of antibodies was controlled for by omitting the primary antibodies from sections that were subsequently incubated with secondary antibodies and TSA. Tissue sections were washed and mounted using Vectashield anti-fade mounting medium containing DAPI (Vector Laboratories) for nuclear counterstaining. Fluorescent images were captured using a Leica DM5500B fluorescence microscope followed by blind 3D deconvolution.

### Inclusion quantification

FUS, TRN1, hnRNP R and hnRNP Q stained slides were scanned using a Leica Slide Scanner SCN400. Regions of interest were digitally marked and ten random fields of view representing a total area of 500 μm^2^ were generated for each case in the frontal cortex and the granule cell layer of the dentate gyrus using ImageJ and a Python script. The number of FUS, TRN1, hnRNP R and hnRNP Q positive inclusions in each field of view were manually quantified. Statistical differences between groups was performed using the Kruskal-Wallis one-way analysis of variance on GraphPad Prism 5 software.

### Biochemical fractionation and immunoblot analysis

Proteins were sequentially extracted using ultracentrifugation in buffers of increasing stringency, adapted from a protocol previously described [27]. To prevent carry over, each extraction step was performed twice. Only supernatants from the first extraction steps were analysed while supernatants from the wash steps were discarded. Tissue samples from frontal cortex (grey matter) were homogenized at a ratio of 1:2 (weight/volume) in high-salt buffer (50 mM Tris-HCl, 750 mM NaCl, 10 mM NaF, 5 mM EDTA) containing 1% Triton-X and protease and phosphatase inhibitors (Roche). Tissue homogenates were initially centrifuged at 1000 g to remove nuclear and membrane debris. The resulting supernatant was subjected to ultracentrifugation at 120000 g for 30 min at 4 °C. The supernatant was retained and termed the high-salt fraction. The pellet was resuspended in radioimmunoprecipitation (RIPA) buffer (50 mM Tris–HCl, 150 mM NaCl, 1% NP-40, 0.5% deoxycholate) containing 2% sodium dodecyl sulphate (SDS) and protease and phosphatase inhibitors. This was then subjected to ultracentrifugation at 120000 g for 30 min at 15 °C, with the resulting supernatant termed the RIPA-SDS fraction. The final pellet was resuspended in 8 M urea containing 8% SDS to become the urea-soluble fraction.

Protein concentrations from each fraction were determined by the bicinchoninic acid protein assay (Pierce) and 20, 20 and 5 μg of protein from the high-salt fraction, RIPA-SDS fraction and urea fractions, respectively, from each case were loaded onto 4–12% Bis–Tris polyacrylamide gels (Invitrogen) and run at 200 V with MES [2-(N-morpholino) ethanesulphonic acid] buffer (Invitrogen) under reducing conditions. Following electrophoresis, the proteins were transferred onto Hybond P membrane (GE Healthcare), blocked with 5% non-fat dried milk in phosphate-buffered saline containing 0.1% Tween and probed overnight with primary antibody diluted in 2.5% bovine serum albumin (Sigma) in phosphate-buffered saline containing 0.1% Tween. Following washes in phosphate-buffered saline-Tween, blots were developed using horseradish peroxidase conjugated anti-rabbit or anti-mouse IgG secondary antibodies (DAKO) and visualized by an enhanced chemiluminescence reaction (Millipore) and the Li-Cor Odyssey imaging system (Li-Cor). The primary antibodies used for immunoblotting were; FUS (Novus aa1–50, 1:4000), hnRNP R (Abcam ab30930, 1:200), hnRNP Q (Millipore 05–1517, 1:500).

## Results

### hnRNP R mRNA expression is increased in some FTLD subtypes

**Fig. 1:**
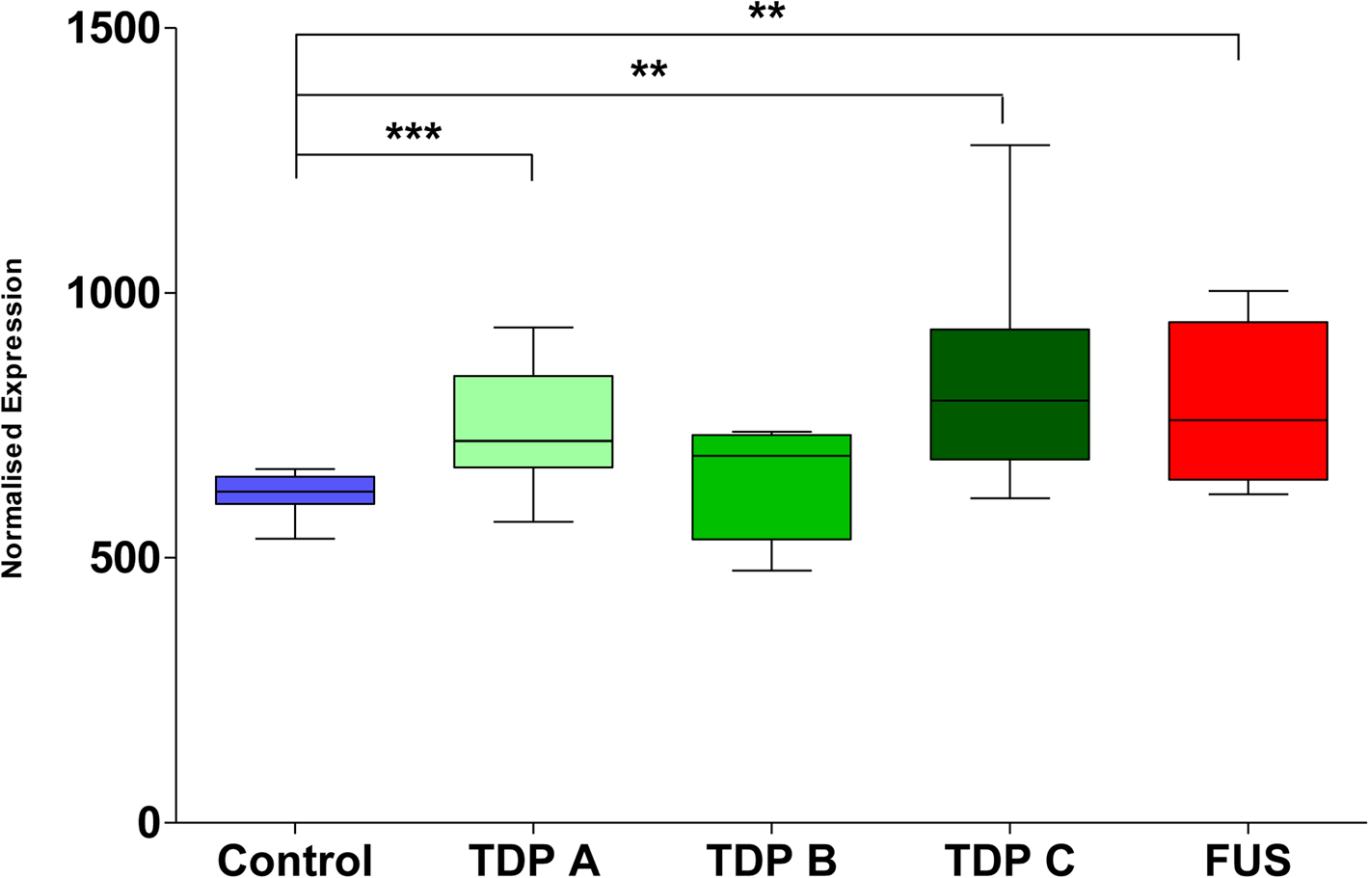
hnRNP R expression is increased in some FTLD subtypes. Nanostring expression analysis of hnRNP R mRNA levels in the frontal and temporal cortices of FTLD-TDP and FTLD-FUS cases compared to neurologically normal controls. A significant increase in expression of hnRNP R was identified in FTLD-TDP A (*p* = 0.0005, *n* = 19), FTLD-TDP C (*p* = 0.0036, *n* = 7) and FTLD-FUS (*p* = 0.0048, *n* = 5) compared to controls (*n* = 6). No significant difference was found between controls and FTLD-TDP B (*p* = 0.5739, *n* = 3). One-way analysis of variance

The mRNA expression of hnRNP R was analysed in the frontal and temporal cortices of FTLD-FUS and FTLD-TDP (Type-A, Type-B and Type-C) cases and compared to expression in the frontal and temporal cortices of neurologically normal control cases with no pathological abnormalities. mRNA expression was analysed using NanoString technology for high-sensitive capture of mRNA transcripts. Normalised expression indicated that hnRNP R mRNA expression was significantly increased compared to normal controls in FTLD-TDP type A (*p = 0.0005*), FTLD-TDP type C (*p = 0.0038*) and FTLD-FUS (*p = 0.0048*) subtypes relative to controls. No significant difference in hnRNP R expression was found between controls and FTLD-TDP type B (*p = 0.5739*) subtype (Fig. 1).

### hnRNP R and hnRNP Q are localised to the nucleus in frontal and hippocampal neurons

**Fig. 2:**
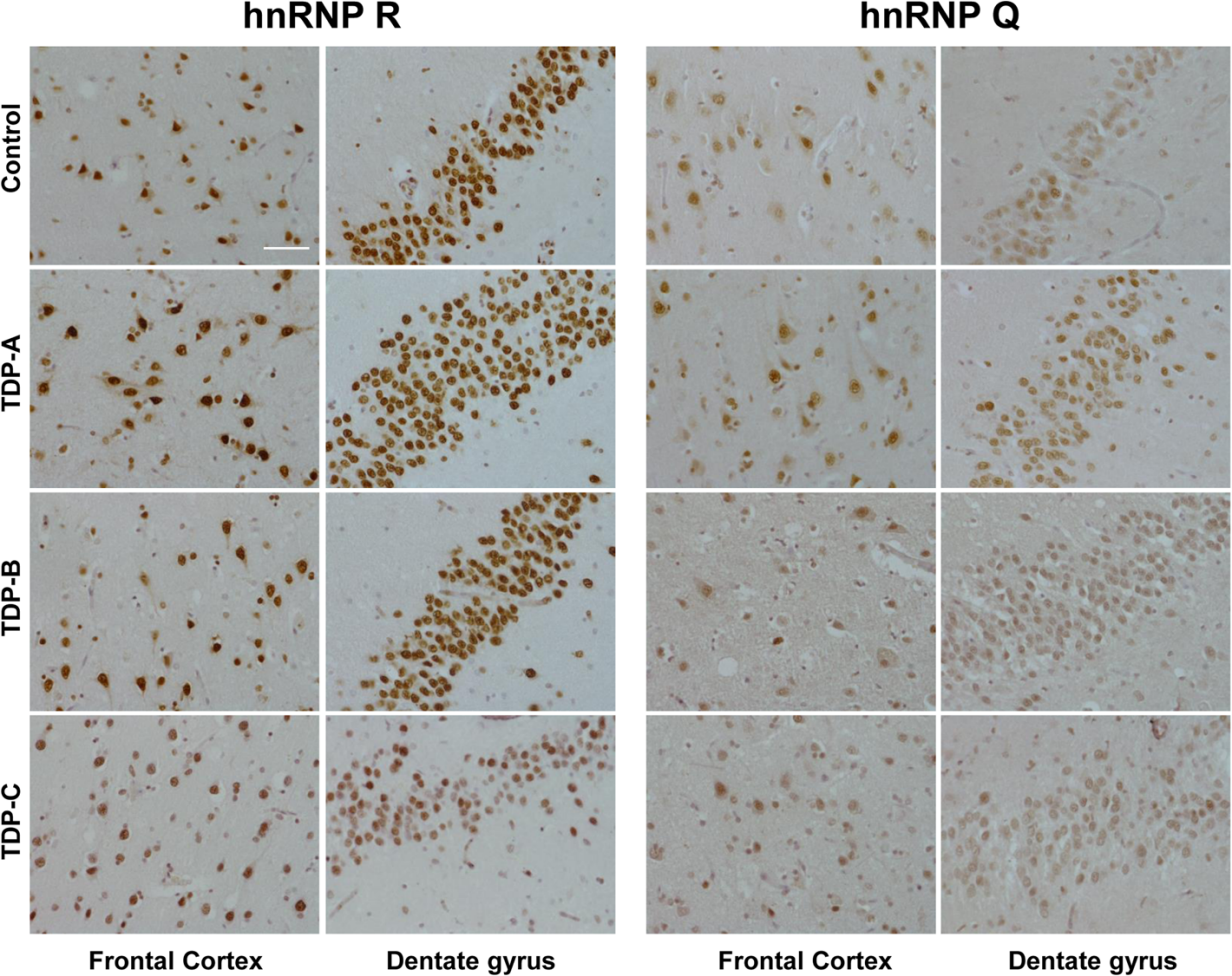
hnRNP R and hnRNP Q localise to the nucleus in the frontal cortex and hippocampus in FTLD-TDP and normal controls. Representative images of hnRNP R and hnRNP Q immunohistochemistry in the frontal cortex and granular cell layer of the dentate gyrus in a neurologically normal control and the FTLD-TDP subtypes (TDP-A, TDP-B, TDP-C and FUS). Strong neuronal nuclear hnRNP R and hnRNP Q staining is observed in both controls and the FTLD-TDP subtypes, with occasional weak cytoplasmic staining in some neurons. Scale bar represents 50 μm in all images

Given that hnRNP R expression was increased in several FTLD subtypes, the cellular distribution of hnRNP R, and the closely related protein, hnRNP Q (Additional file 1: Figure S1), were investigated using immunohistochemical staining in the frontal cortex and hippocampus from FTLD (TDP-A, TDP-B, TDP-C and FUS) cases. Strong neuronal nuclear staining of hnRNP R and hnRNP Q was observed in control and all FTLD subtypes, with occasional neurons additionally showing a weaker cytoplasmic staining pattern. For both proteins, the intensity of the neuronal staining varied among cases and was thought to be due to variation in fixation time; the shorter the fixation time, the higher the intensity of the staining. No pathological inclusions containing hnRNP R or Q were detected in control cases or the FTLD-TDP subtypes (Fig. 2).

### hnRNP R and hnRNP Q are insoluble in FTLD-FUS

**Fig. 3:**
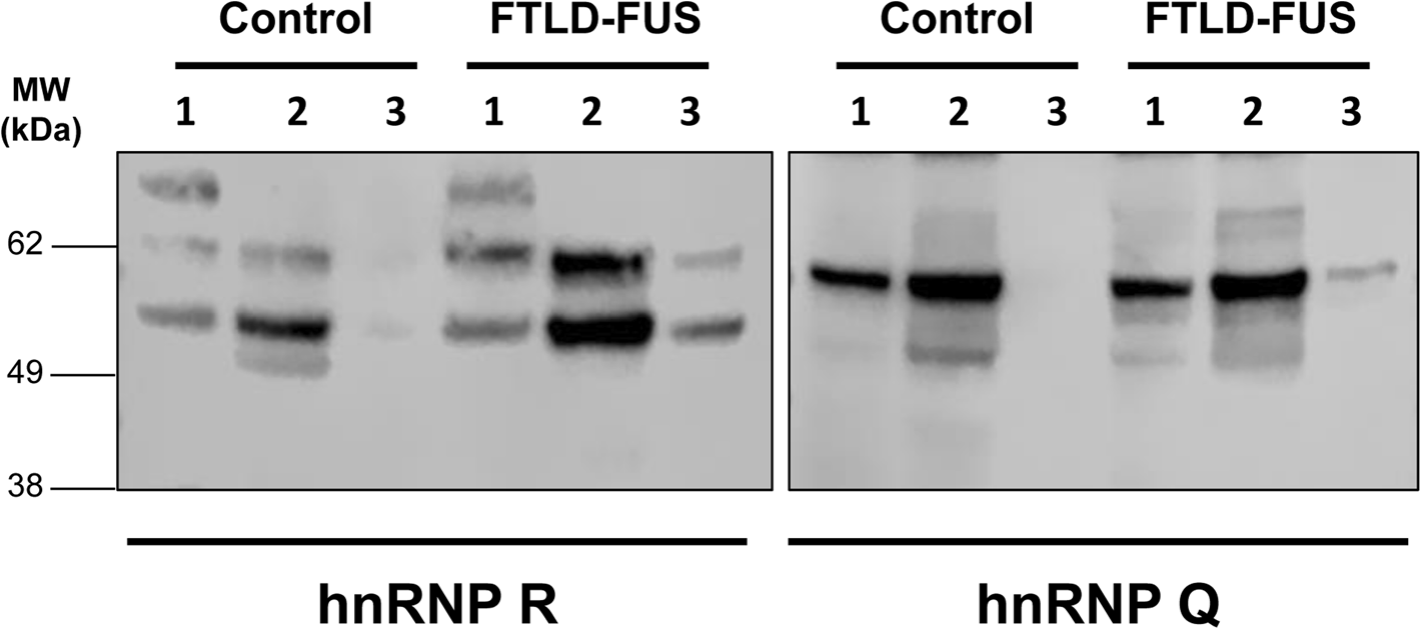
hnRNP R and Q are insoluble in FTLD-FUS. Representative immunoblots demonstrating hnRNP R and hnRNP Q in fractions of varying solubility in FTLD-FUS and normal control brains. Proteins were sequentially extracted into high salt (lane 1), RIPA/ 2% SDS (lane 2), and 8 M urea / 8% SDS (lane 3) fractions. 20 μg of protein was loaded from high salt and RIPA-SDS fractions, while 5 μg of protein was loaded from the urea fractions. Two prominent bands were observed for hnRNP R, whilst a single band was observed for hnRNP Q. Only the FTLD-FUS cases showed bands in the insoluble urea fraction for both proteins

To establish the biochemical characteristics of both hnRNP R and hnRNP Q in FTLD-FUS and normal control brains, protein was sequentially extracted from flash frozen frontal cortex. Buffers containing increasing detergent strength were used to investigate the biochemical fractions with different solubility characteristics. We demonstrated that the two antibodies recognised different proteins on the blots. Although both hnRNP R and hnRNP Q were found in both the soluble and detergent soluble fraction in both FTLD-FUS and the normal controls, urea-soluble hnRNP R and hnRNP Q was only present in FTLD-FUS (Fig. 3).

### hnRNP R and hnRNP Q form inclusions specifically in FTLD-FUS

**Fig. 4:**
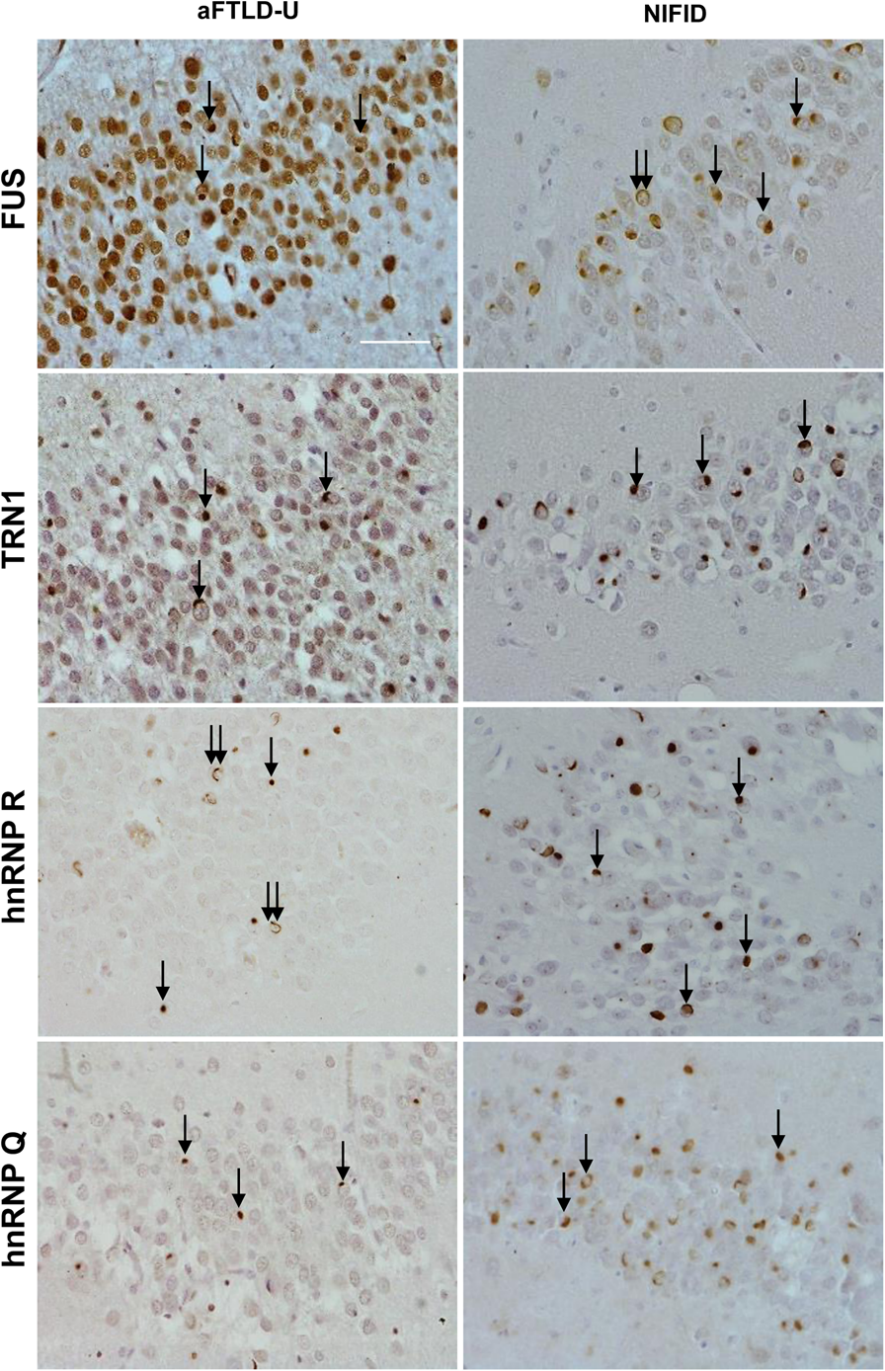
hnRNP R and hnRNP Q form frequent inclusions in FTLD-FUS. Representative images of FUS, TRN1, hnRNP R and hnRNP Q immunohistochemical staining in the granule cell layer of the dentate fascia of the hippocampus in NIFID and aFTLD-U subtypes of FTLD-FUS. Single arrows indicate neuronal cytoplasmic inclusions and double arrows highlight intranuclear inclusions. Scale bars represent 50 μm in all images

Post-mortem brain tissue was available from six NIFID and seven aFTLD-U cases. These were immunohistochemically assessed for hnRNP R and hnRNP Q pathology. All FTLD-FUS cases investigated had abundant hnRNP R and Q immunoreactive neuronal cytoplasmic inclusions, and occasional neuronal intranuclear inclusions, in the frontal cortex and hippocampal granule cell layer (Fig. 4). In some, but not all, neurons in the FTLD-FUS cases, the presence of hnRNP R or Q immunoreactive inclusions depleted the normal nuclear staining of these proteins. However, this was variable between cases, and may reflect variation in tissue fixation time.

**Fig. 5:**
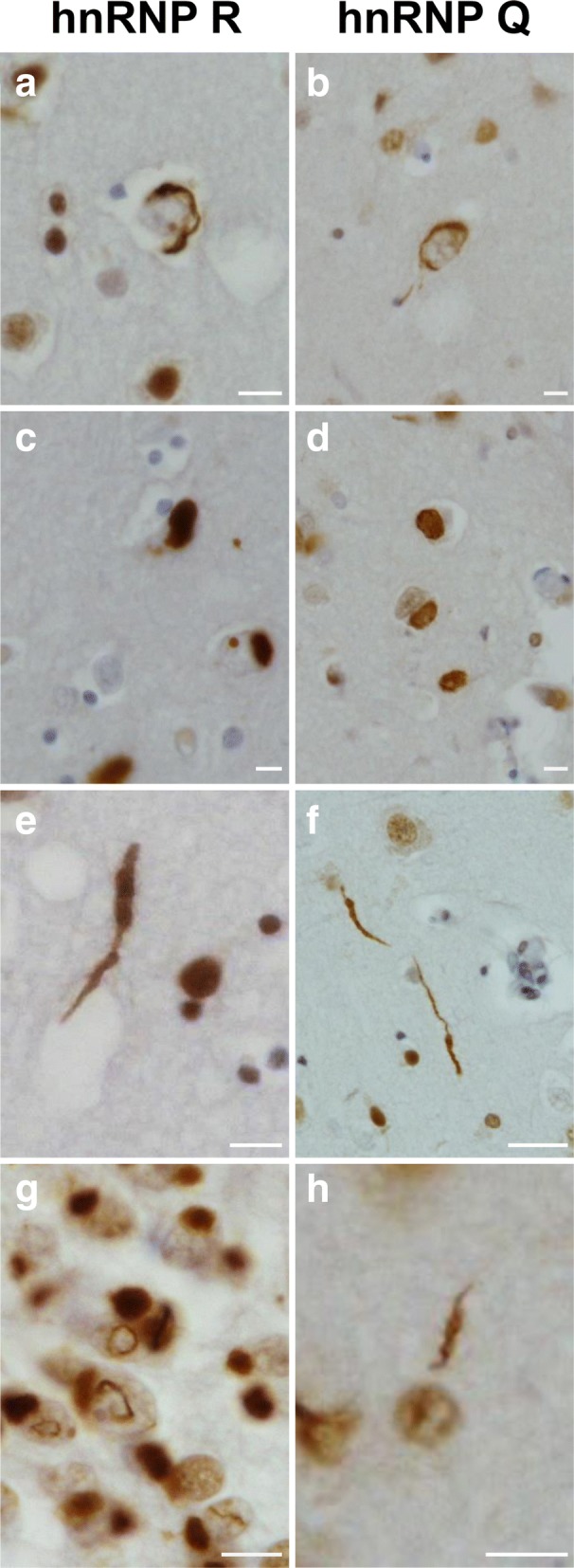
hnRNP R (**a**, **c**, **e** and **g**) and hnRNP Q (**b**, **d**, **f** and **h**) form a range of inclusions types in FTLD-FUS. Immunohistochemical staining demonstrates the different pathological inclusions types found to be immunoreactive with anti-hnRNP R and anti-hnRNP Q antibodies. Crescent neuronal cytoplasmic inclusions (**a** and **b**), bean-shaped neuronal cytoplasmic inclusions (**c** and **d**), dystrophic neurites (**e** and **f**) and intranuclear neuronal inclusions (**g** and **h**). Scale bars represent 10 μm in all images

Between each case, the abundance and distribution of inclusions stained with hnRNP R or hnRNP Q varied but within each case the staining was comparable to FUS and TRN1 immunohistochemistry, which has previously been reported for these cases [6, 27]. As with FUS and TRN1 staining, the NIFID cases consistently showed more hnRNP R and hnRNP Q positive inclusions than aFTLD-U cases. In the hippocampus, hnRNP R and hnRNP Q immuno-reactive neuronal cytoplasmic inclusions were observed in dentate gyrus granule cell layer in both NIFID and aFTLD-U subtypes, although the frequency of inclusions was much higher in the NIFID subtype (Fig. 4). These inclusions were typically bean-shaped or Pick-like structures adjacent to the nucleus, however occasional crescent-shaped inclusions surrounding nuclei were also observed. In some cases, hnRNP R and hnRNP Q neuronal vermiform inclusions were also observed in the granular cell layer in both NIFID and aFTLD-U subtypes. Additionally, crescent-shaped neuronal cytoplasmic inclusions and rod-like structures in neuronal intranuclear inclusions were observed (Fig. 5) as previously described for FUS and TRN1 staining in these cases.

In the frontal cortex, crescent-shaped neuronal cytoplasmic inclusions surrounding the nucleus containing hnRNP R and Q were frequently observed in both NIFID and aFTLD-U cases (Fig. 5a and b), as well as dense, bean-shaped or larger Pick-body-like structures adjacent to the nucleus (Fig. 5c and d). Neuronal intranuclear inclusions were also observed in both FTLD-FUS subtypes, but at a much less frequent rate than neuronal cytoplasmic inclusions. These typically formed a rod-like structure through the nucleus (Fig. 5g and h). Similar to FUS and TRN1 staining, hnRNP R and Q immunoreactive neuronal cytoplasmic and intranuclear inclusions were less frequently observed in the cortex of aFTLD-U cases than NIFID cases. hnRNP R and Q immunoreactive dystrophic neurites were also observed through the cortex of both NIFID and aFTLD-U cases (Fig. 5e and f).

### hnRNP R and hnRNP Q are detected in inclusions as frequently as FUS and TRN1

**Fig. 6:**
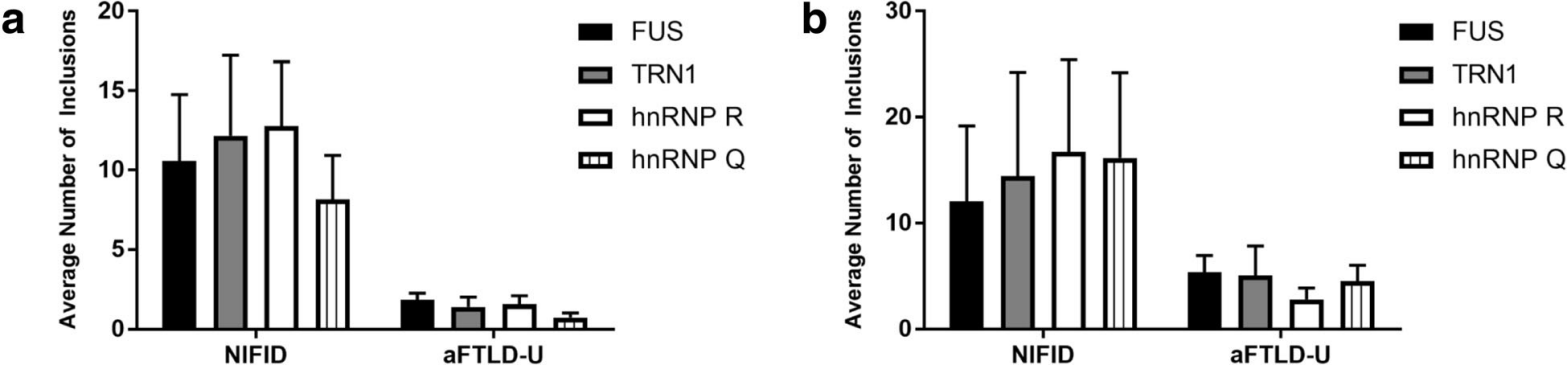
hnRNP R and hnRNP Q inclusions occur as frequently as FUS and TRN1 inclusions. Quantification of FUS, TRN1, hnRNP R and hnRNP Q inclusions in the frontal cortex (**a**) and granular cell layer of the hippocampus (**b**) in NIFID and aFTLD-U cases. No significant differences were found between the number of each inclusion type in either FTLD-FUS subtype in either brain region. Kruscall-Wallis one-way analysis of variance. **a** NIFID cases, *n* = 5, *p* = 0.7978. aFTLD-U cases, *n* = 5, *p* = 0.2856. **b** NIFID cases, *n* = 6, *p* = 0.9723. aFTLD-U cases, *n* = 5, *p* = 0.8934

To quantitatively assess the abundance of pathological inclusions containing hnRNP R and hnRNP Q relative to FUS or TRN1 in FTLD-FUS cases, the number of FUS, TRN1, hnRNP R and hnRNP Q positive inclusions on immunohistochemically stained sections were counted in a defined area of the grey matter of the frontal cortex (Fig. 6a) and in the granule cell layer of the dentate gyrus in the hippocampus (Fig. 6b). Inclusion quantification indicated variation between cases in the number of all inclusions counted, however no statistically significant differences were found between the average number of FUS, TRN1, hnRNP R and hnRNP Q positive inclusions in both NIFID (frontal cortex *p = 0.7978*, dentate gyrus *p = 0.9723*) and atypical FTLD-FUS (frontal cortex *p = 0.2856*, dentate gyrus *p = 0.8934*) subtypes. As expected, the number of FUS, TRN1, hnRNP R and hnRNP Q positive inclusions were consistently higher in the NIFID cases than aFTLD-U.

### hnRNP R and FUS co-localise in inclusions in FTLD-FUS

**Fig. 7:**
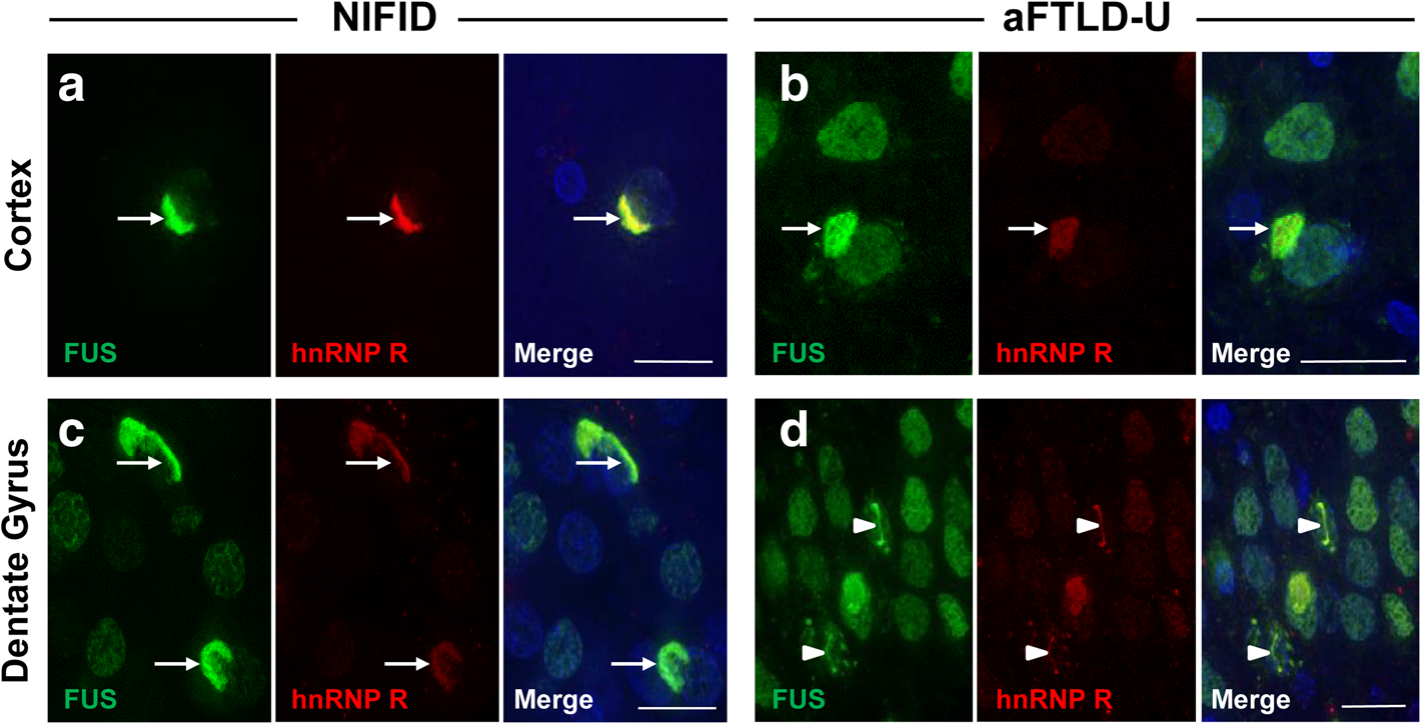
hnRNP R co-localises with FUS inclusions in NIFID and aFTLD-U cases. Representative images of double-label immunofluorescence in the cortex (**a** and **b**) and granular cell layer of the dentate gyrus (**c** and **d**) of a NIFID and aFTLD-U case demonstrating colocalisation of FUS (green) and hnRNP R (red) in neuronal cytoplasmic inclusions (white arrows) and intranuclear neuronal inclusions (white arrow heads). Neuronal nuclei are counterstained with DAPI. Scale bars represent 20 μm in all images

To investigate whether the pathological inclusions labelled with hnRNP R also contained FUS, double immunofluorescence staining was performed on both NIFID and aFTLD-U cases (Fig. 7). Due to the anti-FUS antibody and anti-hnRNP Q antibody being raised in the same species, double immunofluorescence staining with these two antibodies was unable to be performed. Qualitative assessment of the fluorescence images indicated that, in all FTLD-FUS cases assessed, hnRNP R co-localised with FUS in neuronal cytoplasmic and intranuclear inclusions in the cerebral cortex and granule cell layer of the dentate gyrus in the hippocampus, in both NIFID (Fig. 7a and c) and aFTLD-U (Fig. 7b and d) cases.

## Discussion

This study has shown for the first time that hnRNP R and hnRNP Q are mislocalised into pathological inclusions in two subtypes of FTLD-FUS, expanding the spectrum of DNA/RNA binding proteins linked to FTLD pathobiology. mRNA expression analysis revealed that hnRNP R expression was increased in FTLD-TDP A, FTLD-TDP B and FTLD-FUS subtypes but immunohistochemical examination demonstrated that this protein, and the closely related hnRNP Q protein, only show pathological mislocalisation specifically in FTLD-FUS. We have previously shown an increase in mRNA expression of other hnRNP proteins in FTLD-FUS, however these proteins were not identified in the pathological inclusions [17]. The pathological inclusions containing hnRNP R and hnRNP Q in the frontal cortex and hippocampus of these cases had a similar localisation pattern and morphological features to the previously described FUS and TRN1 inclusions [6, 27]. Quantification of hnRNP R and hnRNP Q inclusions indicated they were as frequent as inclusions containing FUS or TRN1, and double-immunofluorescence confirmed the co-localisation of hnRNP R with FUS in both neuronal cytoplasmic and intranuclear inclusions. Additionally, biochemical fractionation demonstrated a shift in the solubility of hnRNP R and hnRNP Q in a similar manner as we previously reported for FUS and TRN1 in the FTLD-FUS cases [6]. These findings reinforce the hypothesis that the pathogenesis of FTLD-FUS extends beyond the FET proteins, known TRN1 cargoes and dysfunctional nuclear import, but rather implicates a broader dysregulation of DNA/RNA binding proteins.

hnRNP R and hnRNP Q are multi-functional, RNA-binding proteins containing three RNA recognition motifs, one acidic rich domain and an RGG domain [21, 38]. Despite being widely expressed in neuronal tissue little is known about these hnRNP in the context of neurodegenerative diseases [44]. The two proteins have high sequence homology with sequence alignment of their canonical isoforms showing that they are 81.2% identical at the amino acid level [37]. Consequently, they are known to have similar, but distinct, functional roles within the cell [8, 19, 37]. hnRNP R is involved in axonal RNA transport and processing, the expression of immunity factors, and transcription and degradation process of *c-fos* mRNA [11, 16, 23, 49], whilst hnRNP Q, also known as SYNCRIP, is implicated in the maintenance of circadian rhythms and be involved in the regulation of mRNAs responsible for neuronal morphogenesis [10, 25, 31]. Both proteins are known to interact with the survival motor neuron (SMN) protein [1] and be involved in pre-mRNA splicing as components of the spliceosome [9, 38, 51, 56]. Recent analysis of these proteins in a cellular model has found them to be important regulators of neuronal homeostasis and indicated that their disruption could impair distinct pathways in the central nervous system axis [8]. Interestingly, a link between TDP-43 and hnRNP Q has previously been reported as hnRNP Q is capable of rescuing TDP-43 toxicity in *Drosophila melanogaster* model [3], whilst significant alterations in hnRNP Q were found in ALS compared controls [4]. In contrast, no interactions have previously been reported between FUS and hnRNP R or hnRNP Q.

A prominent hypothesis to explain the pathogenesis of FTLD-FUS is that pathological aggregation of FUS and other FET proteins results from an impaired interaction with their nuclear importer, TRN1 [34, 43]. It is believed that this may be a result of impaired methylation of arginine residues in the RGG3 domains of the FET proteins, which causes overly tight binding of the FET proteins to TRN1. A consequence of this aberrant binding is lack of dissociation of the FET-TRN1 complex once inside the nucleus, resulting in the re-export of the complex and accumulation of FET proteins and TRN1 in the cytoplasm [12, 13]. Recent work has also shown that aberrant arginine methylation of FUS, as seen in FTLD-FUS patients, promotes the phase transition of FUS into liquid-like droplets which form solid, fibrous aggregates over time, promoting their pathological aggregation [22, 47]. Given the structural and functional similarity between the FET proteins, it is possible that arginine methylation may have a similar effect on EWS and TAF15, although this remains to be investigated. Whilst this hypothesis can explain the presence of TRN1 and the three FET proteins in pathological inclusions in FTLD-FUS, it cannot explain the pathological accumulation of non-FET proteins, such as hnRNP R, hnRNP Q and the other hnRNP proteins previously identified in these inclusions [17]. With the exception of hnRNP A1 and hnRNP D, the majority of these proteins are not predicted to be imported by TRN1 [30, 45, 53], and it is unclear to what extent these proteins are capable of liquid-liquid phase separation or subject to the effects of arginine methylation. This suggests that it is not only the FET proteins that are responsible for FTLD-FUS pathology but rather implicates dysfunction in a broader spectrum of RNA binding proteins. Unlike EWS, TAF15 and other hnRNPs, which are only found in a proportion of FUS inclusions, quantification of hnRNP R and hnRNP Q inclusions revealed that these proteins are found as frequently as FUS and TRN1 in inclusions. This suggests these proteins have a central role in the pathogenesis of FTLD-FUS, however it is currently unclear, whether the accumulation of these proteins is a trigger or consequence of FUS aggregation.

FUS, hnRNP R and hnRNP Q are all RNA binding proteins that have a wide range of functions linked to various aspects of mRNA metabolism. They have all been reported to bind to the SMN protein spliceosome complex and are all known to shuttle between the nucleus and cytoplasm associated with mRNAs [38, 51, 57]. This indicates these proteins have similar functions and could suggest that they interact with each other under physiological conditions within the cell. One hypothesis to explain the co-aggregation of FUS, hnRNP R and hnRNP Q could be that these proteins associate with each other in a protein-RNA complex, either directly via a protein-protein interaction, or indirectly by binding to the same mRNA transcripts. If the proteins form part of the same complex, then aberrant aggregation of one of the proteins could trigger the co-deposition of the associated proteins. It may also be possible that FUS and hnRNP R and hnRNP Q do not interact with each other physiologically and localise to pathological aggregates independently. Endogenous FUS and hnRNP Q have both been shown to localise to cytoplasmic stress granules under specific cellular stress conditions [48, 52]. It may therefore be possible that these proteins co-localise in stress granules only during cellular stress and it is the aberrant disassembly of these granules that results in co-aggregation of these proteins in pathological inclusions. Several groups have proposed that the pathological accumulation of FUS, and other ALS/FTD linked proteins, is initiated by their assembly in stress granules or other RNA granules [5, 14, 32]. The mechanism by which FUS condenses into stress granules by liquid-liquid phase separation is well-characterised and is known to be driven by cation-pi interactions between tyrosine in its N-terminal LCD domain and arginine residues in the C-terminal RGG domain [22, 47]. Whether hnRNP R and hnRNP Q possess similar phase transitioning properties that enable them to condense into stress granules is currently unknown and requires further investigation. Both hnRNP proteins contain C-terminal RGG domains but only hnRNP R is predicted to have a LCD domain.

The identification of hnRNP R and/or hnRNP Q in pathological inclusions in FTLD-FUS provide two candidate genes for genetic screening in FTLD. All FTLD-FUS cases used in this study have previously been screened for mutations in a variety of genes linked to FTD and ALS [27, 50], but to date, no genetic mutations have been identified as causative of FTLD-FUS. Screening the *HNRNPR* and *HNRNPQ* genes for mutations in these cases could identify mutations linked to disease. It would also be interesting to screen for mutations and assess hnRNP R and hnRNP Q pathology in ALS-FUS cases. To date, none of the additional proteins identified in FTLD-FUS inclusions have been found in ALS-FUS inclusions [43]. This is hypothesised to reflect the differing pathogenic mechanisms of the diseases, however, the end-point in both diseases is the pathological aggregation of FUS and it is possible that other proteins associated with FUS will also be common to both diseases. ALS-FUS cases should therefore be assessed for hnRNP R and hnRNP Q pathology to determine whether the dysregulation of these proteins is specific to FTLD-FUS or is a common feature shared by FUS pathologies.

In summary, the identification of hnRNP R and hnRNP Q in pathological inclusions in the FTLD-FUS cases adds two new proteins to the growing list of RNA binding proteins implicated in the pathogenesis of FTLD. Disease causing mutations in TDP-43, FUS, hnRNP A1, hnRNP A2B1, MATR3 and TIA1 all point to disturbed function of RNA binding proteins, especially hnRNPs, as playing a role in the pathogenesis of FTD and ALS [46]. The accumulation of these proteins in cytoplasmic and intranuclear neuronal inclusions was found to be specific to FTLD-FUS cases, although increased hnRNP R mRNA expression was also seen in several FTLD-TDP subtypes. These inclusions were found to co-localise with and occur as frequently as inclusions containing FUS, suggesting these proteins may play a role in the pathogenesis of FTLD-FUS. The relationship between FUS and these hnRNP proteins has not been previously explored and future experiments should be performed to establish whether these proteins directly or indirectly associate with FUS as this may help to establish the mechanism by which these proteins co-aggregate. Future biochemical experiments are also required to address whether it is both hnRNP R and hnRNP Q accumulating in these inclusions because the high level of homology between these proteins has made this difficult to decipher by immunohistochemical methods. Further functional understanding of these two new RNA binding proteins in FTLD-FUS aggregates may help to elucidate the mechanism by which these inclusions form and reveal novel functions for these hnRNP proteins.

## Additional file

Additional file 1: **Figure S1.** Schematic diagram of hnRNP R and hnRNP Q proteins including antibody binding sites and sequence alignment. The schematic diagram illustrates the structural similarities between hnRNP R and hnRNP Q. The antibody binding sites for the antibodies used in this study are shown on the diagram (a). Protein sequence alignment showing similarities between hnRNP R and hnRNP Q sequences (b). Abbreviations: Acidic – Acidic rich domain; RRM – RNA recognition motif; RGG – Arginine and glycine rich domain; NLS – Nuclear localisation signal; QN – Glutamine and asparagine rich domain. (TIF 3258 kb)
