# Supplementary figures and images for: Heterogeneous nuclear ribonucleoproteins R and Q accumulate in pathological inclusions in FTLD-FUS
Source: Acta Neuropathol Commun. 2019 Feb 12;7:18. doi: 10.1186/s40478-019-0673-y (PMC6371513; doi:10.1186/s40478-019-0673-y)

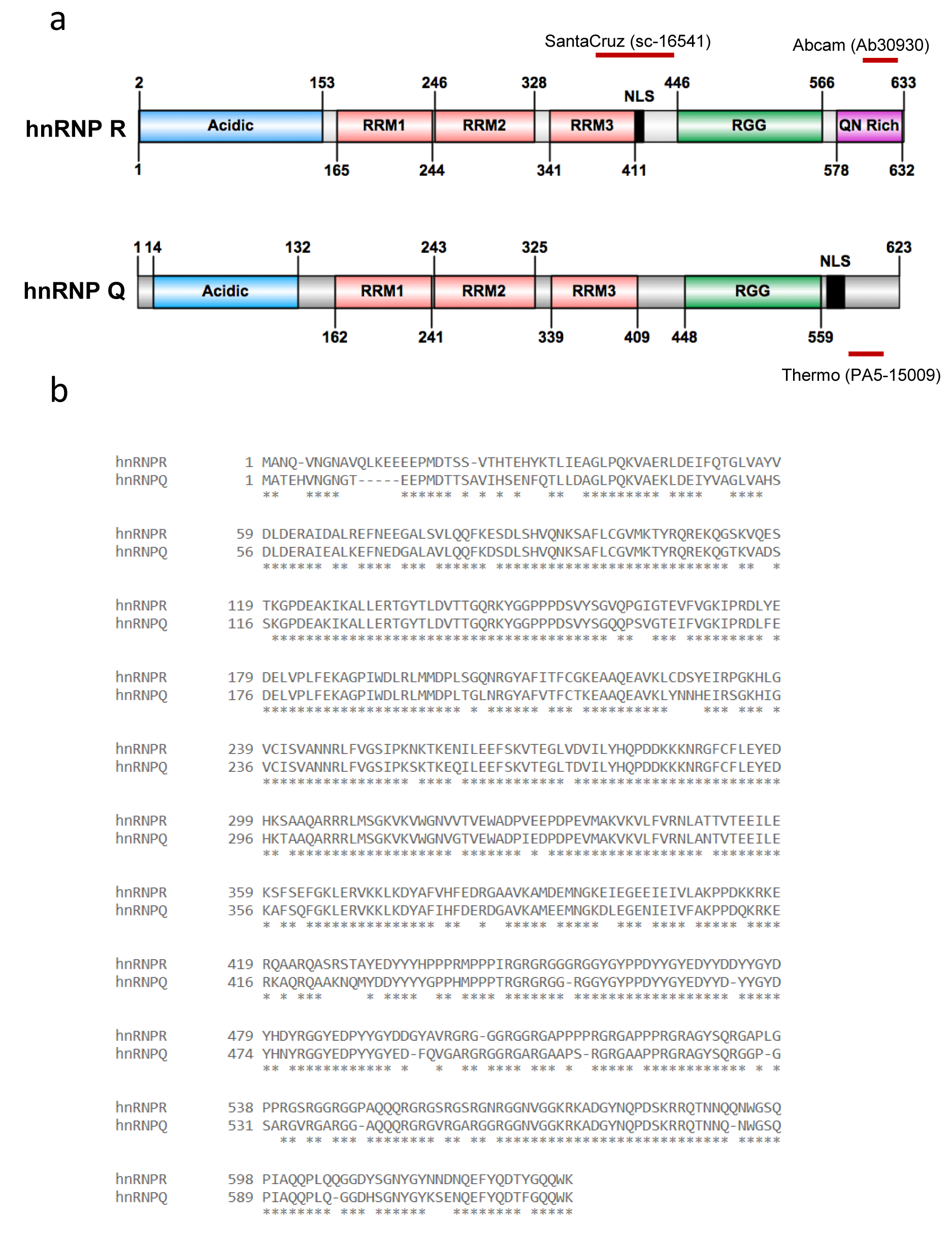

Supplement: Supplementary file 1 — Figure S1. Schematic diagram of hnRNP R and hnRNP Q proteins including antibody binding sites and sequence alignment. The schematic diagram illustrates the structural similarities between hnRNP R and hnRNP Q. The antibody binding sites for the antibodies used in this study are shown on the diagram (a). Protein sequence alignment showing similarities between hnRNP R and hnRNP Q sequences (b). Abbreviations: Acidic – Acidic rich domain; RRM – RNA recognition motif; RGG – Arginine and glycine rich domain; NLS – Nuclear localisation signal; QN – Glutamine and asparagine rich domain. (TIF 3258 kb) [file 40478_2019_673_MOESM1_ESM.tif]
